# Performance of Optical Coupling Materials in Scintillation Detectors Post Temperature Exposure

**DOI:** 10.3390/s20216092

**Published:** 2020-10-27

**Authors:** Gregory Romanchek, Yuli Wang, Harsha Marupudi, Shiva Abbaszadeh

**Affiliations:** 1 Nuclear, Plasma and Radiological Engineering, University of Illinois at Urbana-Champaign, Urbana, IL 61801, USA; romanch2@illinois.edu (G.R.); smarup2@illinois.edu (H.M.); 2 Electrical and Computer Engineering, University of California, Santa Cruz, CA 95064, USA; ywang812@ucsc.edu

**Keywords:** optical coupling materials, temperature exposure, energy resolution, ultraviolet-visible (UV-Vis) spectra experiments

## Abstract

In this paper, the room-temperature performance of different optical coupling materials post temperature exposure was tested. The tested couplers included OC431A-LVP, OG0010 optical grease, BLUESIL V-788, and SAINT-GOBAIN BC-630. This was done by subjecting the whole detector with newly applied optical coupling materials to a 2-h temperature exposure—ranging from −20 to 50 °C and then by letting it return to room temperature before collecting a spectrum from a Cs-137 source. The energy resolution at 662 keV was computed as the metric for evaluating the performance. Three trials were run at each coupler–temperature combination. Our results reveal that the performance of all coupling agents do indeed change with temperature after the 2-h exposure. Over all the tested temperature trials, the energy resolution ranged from 11.4 to 14.3% for OC431A-LVP; 10.2 to 14.6% for OG0010; 10 to 13.4% for BLUESIL V-788; and 9.8 to 13.3% for SAINT-GOBAIN BC-630. OC431A-LVP had the lowest variance over the full range, while BC-630 was the most constant for temperatures above 20 °C. Ultraviolet-visible (UV-Vis) spectra experiments were also performed on isolated optical coupling materials to measure the light absorption coefficient. The results show that the temperature-induced variance in light absorption coefficient of each optical coupling materials is one of the reasons for the variance in energy resolution performance. Our findings suggest the need for further investigation into this effect and the recommendation that optical coupling materials need to be selected for the task at hand with greater scrutiny.

## 1. Introduction

Scintillation crystals coupled with silicon photomultipliers (SiPMs) are of significant interest for use in radiation detection due to their compact size and high quantum efficiency [1]. Incoming high-energy photons (x-rays and gamma rays) interact with the scintillation crystal, causing emission of scintillation light (optical photons). This light then passes from the scintillation crystal to the SiPM (photosensor) through a surface interface commonly filled with optical coupling material, also referred to as optical grease or gel. For radiation detectors operating over a range of temperatures, such as mobile detectors [2], having consistent or predictable performance is vital to the overall functionality of the detector. Temperature changes also arise from heat generated during operations from the read-out electronics [3], leading to special care for the temperature sensitivity of the silicon photomultiplier [4]. An additional component which may require similar attention is the optical coupling material which joins the SiPM and scintillator crystal surfaces.

The goal of optical coupling materials is to provide a medium for optical photons to pass through the scintillator crystal to the SiPM by maintaining an index of refraction similar to that of the scintillation crystal and by providing flush surface contact [5,6]. Mismatching of the index of refraction or poor surface contact can contribute to elevated scattering at the crystal–SiPM interface, resulting in fewer optical photons detected and reduced detector performance [7]. Thus, optical coupling materials must be chosen based on specific material properties which are assumed to be constant throughout deployment. Temperature exposure, however, may alter the long-term material properties of the optical coupler or its surface coverage and thus worsen detector performance. Such changes can include the index of refraction drifting away from that of the scintillator crystal or increased attenuation of optical photons. For mobile detectors, these temperature ranges can be large, repeated, and sudden, though all detectors experience some heating (and subsequent cooling) during operation on the crystal–SiPM surface. However, to the best of our knowledge, the effect of this temperature wear on optical couplers has not been investigated as thoroughly as in the SiPM temperature-dependent performance.

This work aims to investigate the baseline (room-temperature) performance change of common optical coupling materials after short-term temperature shifts. First, the whole detector with new optical coupler is subjected to a single temperature exposure. Here, energy resolution is used as a metric of performance to compare between varies temperature points and the different optical couplers tested. Various common optical coupling materials are used, keeping the remainder of the detector components constant between trials to capture the contribution from coupling material response. Secondly, an ultraviolet-visible (UV-Vis) spectra experiment is also performed on isolated optical coupler samples. This is done to quantify the shifts in light absorption after a single temperature exposure.

## 2. Materials and Methods

### 2.1. Coupling Agents and Detector Crystal

In this paper, OC431A-LVP (SmartGel), OG0010 optical grease, BLUESIL V-788 (Elkem), and BC-630 (SAINT-GOBAIN) are chosen as the candidate optical couplings for our experiments. These are either commonly used in optical devices and gamma detectors [8,9,10] or, in the case of OG0010, are included in spectroscopy kits. Among these coupling materials, BC-630 is the most common for Lutetium-yttrium oxyorthosilicate (LYSO)-SiPM in positron emission tomography [10,11,12]. Table 1 lists the characteristics of all four coupling materials. Please note that BC-630 passed its expiration date of June 2018 during testing. Correspondence with Saint-Gobain support states that “Elevated storage temperatures or longer period of storage will result in slow softening. Despite softening, other properties are unaffected and the product may be used for its intended purpose.” Testing was concluded on Bluesil V-788 before the printed expiration date and had a similar statement regarding longer-term usability in its technical data sheet [13].

Lutetium-yttrium oxyorthosilicate (LYSO) scintillation crystal was chosen as the scintillation crystal for this study due to its high sensitivity on radiation detection [14] and relatively stable performance with different temperatures (i.e., LYSO maintains stable light output as temperature fluctuates) [15]. Keep in mind all readings are taken at room temperature. The properties of LYSO crystal, including density, energy resolution, index of refraction, and effective Z number, are summarized in Table 2. The LYSO(Ce) crystal with affixed reflector was produced by Hilger Crystals [16]. During the individual experimental trials, each coupling agent was tested over the selected temperature range and was paired to a LYSO crystal. The LYSO crystal was covered on all sides with a reflector except for the SiPM face in our tests.

### 2.2. Experiment Setup

**Figure 1:**
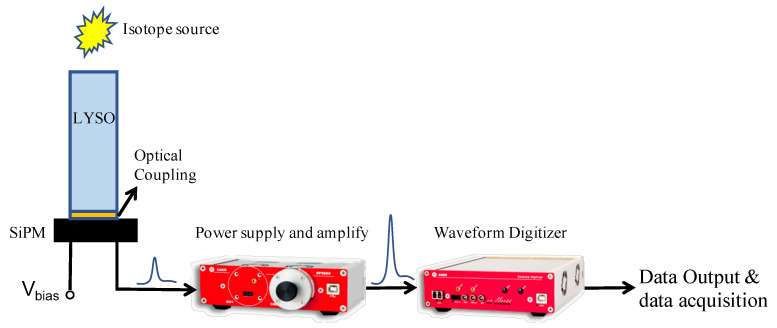
Experimental setup for testing the performance of different optical coupling.

The layout of the experiment setup is shown in Figure 1. Characteristic light output of the samples was measured using a Hamamatsu S13360-6050CS SiPM. The LYSO crystals were irradiated with a 30 μCi Cs-137 source placed in the source holder built into the spectrometer at a fixed distance from the scintillation crystal. Pulses were amplified by a commercial power supply and amplification unit (CAEN SP5600) and subsequently were digitized by a digitizer (CAEN DT5730). Optical coupling material and exposed temperature are the only variables throughout the experiments as other components were kept the same. All digitized waveforms were processed and analyzed by data acquisition software.

### 2.3. Selection of Temperature Exposure Criteria

The temperature range, temperature exposure times, and experimental protocol are generally based upon the American National Standards Institute (ANSI) temperature standards and performance criteria for mobile radiation detectors [22]. ANSI’s required test range is from −30 to 55 °C; therefore −20, −10, 0, 10, 20, 30, 40, and 50 °C were chosen as the experimental temperatures in this paper, which cover the temperature operation conditions of most mobile radiation detectors. A 2-h exposure to the constant temperatures was also chosen based on the ANSI criteria. The detector temperature exposure was sudden as opposed to the gradual heating/cooling recommended by ANSI, however, to better capture real-world usage of mobile detectors (e.g., being moved from a cool vehicle/lab to a hot, outdoor environment). Metrics were gathered after the detector returned to baseline temperature to measure the long-term effects of optical coupler temperature exposure rather than at-temperature performance. The H2200-Heating and Cooling Incubator (Benckmark MyTemp) served as the temperature chamber for 10 to 50 °C, whereas a Portable Compressor Freezer (ACOPOWER) was used for −20 to 0 °C.

### 2.4. Experimental Procedures

Each trial began by selecting the optical coupling material of interest and by cleaning the free face of the crystal and SiPM with ethanol. The temperature chamber was set to the desired temperature and left to equalize to that temperature. The coupling agent was then applied to the crystal and joined to the SiPM according to the manufacturer instructions when available (approximately 1 mm thick for all couplers). Immediately after this assembly, the whole detector apparatus, containing the scintillation crystal, optical coupler, SiPM, and readout board, was then placed in the temperature bath for a 2-h exposure period. The detector was then removed from the chamber and left to stabilize for 20 h back to room temperature. A 10-min spectra of a 30 μCi Cs-137 source placed in the source holder was collected. The source photopeak at 662 keV was fit to a Gaussian distribution, and the energy resolution at 662 keV was computed. Three trials were repeated for each optical coupling material and for each temperature point to capture any variance which may occur, including variable coupling material thickness, nonuniformity, and deviations in ambient temperature or humidity. Optical coupling material was newly applied for every trial and was only tested once, at a single temperature point, before replacement.

In order to verify the consistency of our methodology and to test the overall variance of temperature responses of each coupler, five additional 10-min spectra were collected for each of the optical coupling materials at room temperature: 23 °C. The coupling material was replaced in between each of the five trials for each of the couplers. Here, the 2-h exposure and 20-h wait were omitted because the couplers and detector are stored at room temperature, instead of collecting the spectra immediately. Energy resolution at 662 keV was then computed for each separate spectrum, and the sample variance across these trials was computed for each optical coupler.

The room-temperature variance for each optical coupler was then compared against the variance of the full set of measurements for that coupler—all energy resolution metrics for all trials at each previously tested temperature (24 data points per coupler). This helps us determine two things. First, if the methodology is consistent, the room-temperature variance will be small. Second, if the variance at room temperature for each coupler is much smaller than its total variance over all tested temperatures, then it is unlikely the observed changes in performance are due to methodological inconsistencies.

### 2.5. UV-Vis Spectra Measurements

To further investigate and quantify the reason for variance in optical coupling material performance when exposed to different temperatures, a UV-Vis spectra measurement [23] was performed to explicitly measure the light absorption coefficient of optical coupling materials after exposure and subsequent return to room temperature. The targeted wavelength of UV-Vis measurement was set between 350 nm to 650 nm, which corresponds to the induced light spectrum range of LYSO crystal [24].

UV-Vis experimental procedures start by selecting one optical coupling material of interest. A graduated pipette was used to transfer 1.875 mL of optical coupling material to a 75 mm × 25 mm clean glass glide (from Fisher Scientific Company, Waltham, MA, USA). An experimental spatula was used to evenly spread the optical coupling material to the whole glass slide and to help us obtain a relatively flat and smooth surface of the applied optical coupling material. A micrometer was used to measure the thickness of the optical coupling material and to make sure the thickness over the whole glass slide was 1 mm. The preparation process was the same for the four kinds of optical coupling materials. One trial was measured for each optical coupling material and for each temperature point.

Using the same criteria presented in Section 2.3, the temperature chamber was set to the selected temperature of interest. For each temperature trial, four different optical coupling materials attached with glass slides and one blank glass slide were placed in the temperature chamber for 2-h exposure and were then left to return to room temperature for 20 h. A Cary 60 UV-Vis Spectrometer (Agilent Tech., Santa Clara, CA, USA) was first used to detect the light absorption coefficient of the blank glass slide, the results of which were regarded as the correction for the following measurements. After that, the absorption coefficient of each optical coupling material with glass slides was measured by the UV-Vis Spectrometer based on the results of blank glass slide. The results of the UV-Vis experiments are shown in Section 3.4.

## 3. Results

### 3.1. Experimental Results of Energy Resolution

**Figure 2:**
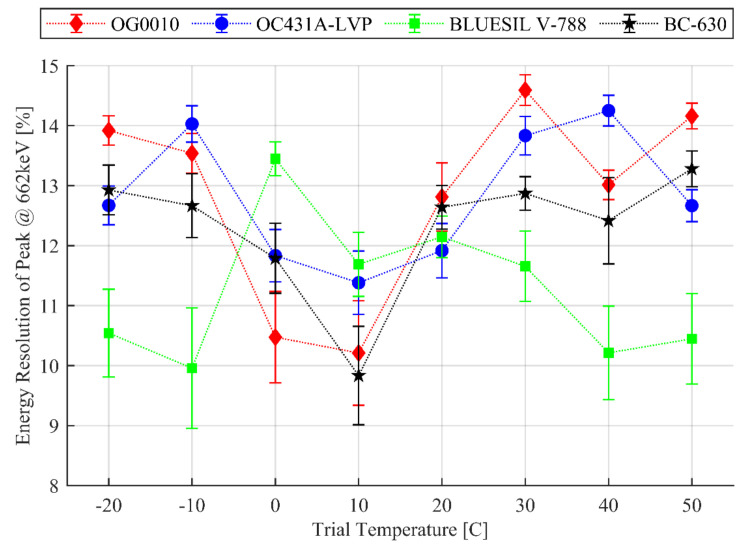
Energy resolution after exposure to the given temperature and return to room temperature using LYSO as the scintillation crystal and coupling agents given in Table 1: The coupling material was newly applied for each temperature point.

**Figure 3:**
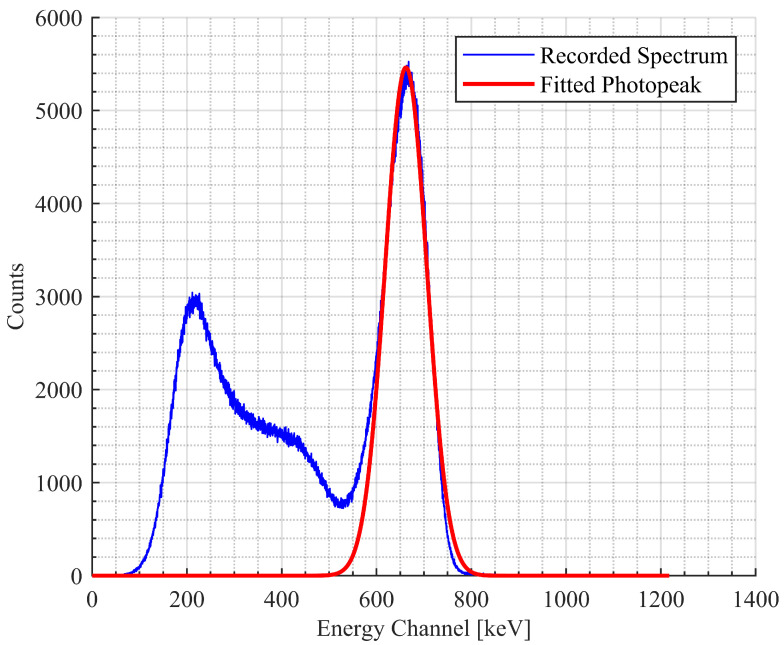
A gamma-ray spectrum from a 10-min collection of a Cs-137 source is shown in blue. The LYSO scintillation crystal and silicon photomultipliers (SiPM) were coupled with Bluesil V-788. This collection was taken after the whole detector was subjected to a 2-h 10 °C temperature bath and a 20-h return to room temperature. A Gaussian fit of the 662 keV photopeak is displayed in red.

The energy resolution results are presented in Figure 2 with error bars corresponding to the standard deviation of the three trials conducted at each temperature for each optical coupler. The goodness of fit of the Gaussian fit remained greater than 0.98 for all trials with a mean and sample standard deviation of 0.9958±0.0042. There was no significant deviation from Gaussian shape. Sample spectra can be seen in Figure 3. Over all the tested temperature trials, the energy resolution ranged from 11.4 to 14.3% for OC431A-LVP; 10.2 to 14.6% for OG0010; 10 to 13.4% for BLUESIL V-788; and 9.8 to 13.3% for SAINT-GOBAIN BC-630.

The agents OG0010, OC431A-LVP, and BC-630 all have similar responses to temperature with optimal energy resolution after exposure to 10 °C and tapering up to 13 to 14% at either temperature extreme. The BLUESIL V-788 displayed the opposite behavior to that of the other couplers: it had worse performance at mid-range temperatures from 0 to 30 °C than at the temperature extremes. It also displayed greater variability in performance at the temperature extremes as noted by observing the error bars. This is odd given how narrow its recommended temperature range is.

It is important to reiterate that these data are not collected at the displayed temperature but instead after exposure to that temperature and return to room temperature. Also, optical coupling material was newly applied for each temperature, so no cumulative effects occurred.

### 3.2. Results for Variance in Temperature Response

The room-temperature variance and the variance of all temperature measurements for each optical coupling material are presented in Table 3. We can immediately observe that the variance for the room-temperature set is much smaller than that of the full set. Thus, we can conclude, first, that the spread of energy resolution data (observable in Figure 2) over the tested temperature range cannot be explained by methodological inconsistency and, second, that the temperature does impact the performance of the detector. Here, we can also see that OC431A-LVP has the smallest overall variance for the tested coupling material at 1.501. This is in agreement with what can be observed in Figure 2. For selected temperature ranges, however, BC-630 performs the most consistently for 20 °C and up.

### 3.3. Experimental Results of Total Count and Photopeak Counts

**Figure 4:**
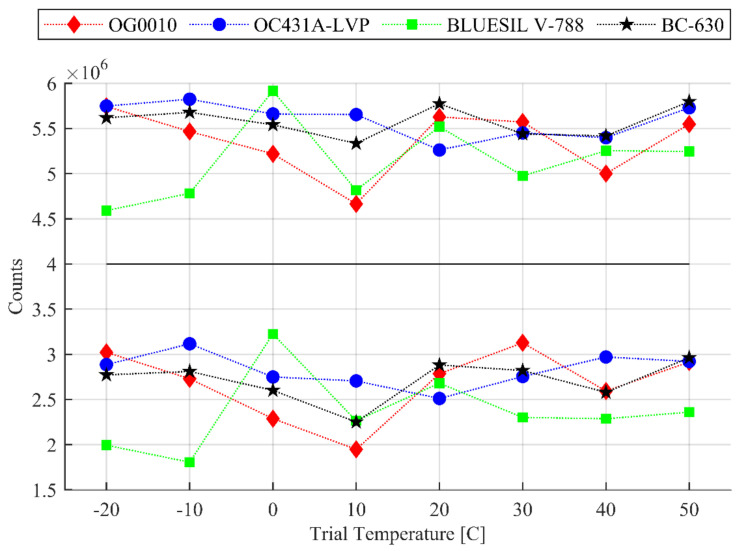
Total (**top**) and peak (**bottom**) counts at the given temperatures using LYSO as the scintillation crystal and coupling agents given in Table 1.

The total count and photopeak counts (counts falling within the Gaussian fit about 662 keV) are shown in Figure 4, and the overall counts statistics are presented in Table 4. The counts collected during the trials display a similar trend to energy resolution. Over all the tested temperature trials, the total counts ranged from ($\times 10^{6}$) 5.26 to 5.80 for OC431A-LVP; 4.70 to 5.80 for OG0010; 4.60 to 6.00 for BLUESIL V-788; and 5.30 to 5.65 for SAINT-GOBAIN BC-630. When we compare the performance results of the total counts, OC431A-LVP and BC-630 had similar small variance over the −20 to 50 °C testing range while BC-630 had the most stable performance above 20 °C.

Notably, again, the agents OG0010, OC431A-LVP, and BC-63 shared similar trends over the temperature range whereas BLUESIL V-788 varied greatly. The variance in counts cannot be attributed to only the natural variance of source intensity and counting statistics.

### 3.4. Experimental Results of UV-Vis Measurements

**Figure 5:**
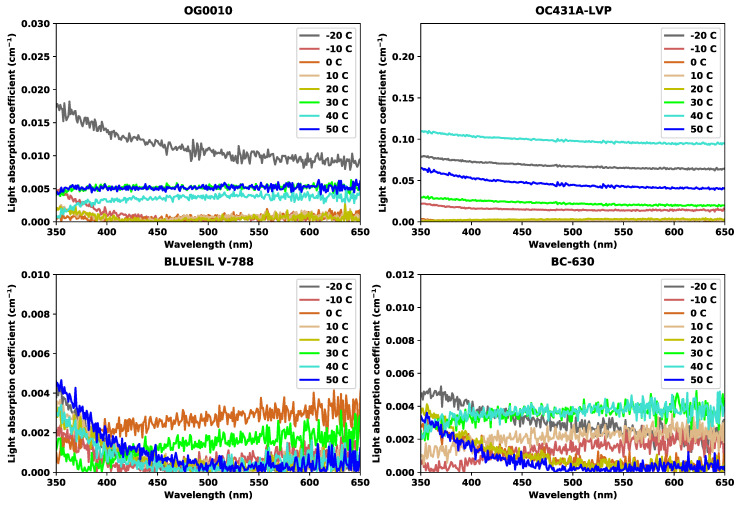
Results of light absorption coefficient (cm${}^{-1}$) range from 350 nm to 650 nm wavelengths at 8 temperature trials for OG0010 (**upper left**), OC431A-LVP (**upper right**), BLUESIL V-788 (**bottom left**), and BC-630 (**bottom right**).

The results of light absorption coefficient for the given temperature trials across 350 nm to 650 nm wavelengths for the four targeted optical coupling materials are shown in Figure 5. Generally, the lower the absorption curve, the better light passes through the optical coupling materials. It is first apparent that the light absorption coefficient for all the tested optical couplers is affected by a single temperature exposure. It is then apparent that each optical coupler has a different response to the temperatures, both in terms of magnitude of response and to which temperature had what affect. Compared to the results of BLUESIL V-788 and BC-630, OG0010 and OC431A-LVP have much smoother and lower noise experimental results regarding the light absorption coefficient. The temperature trials that could obtain the maximum value in light absorption coefficient for the tested optical coupling materials are −20 °C (OG0010), 40 °C (OC431A-LVP), 0 °C (BLUESIL V-788), and 30 °C (BC-630).

**Figure 6:**
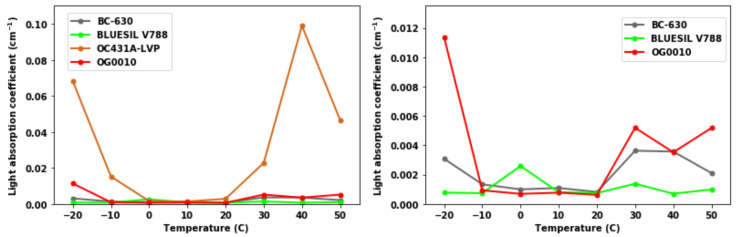
Results summary of UV-Vis experiments at given temperature trials.

To better interpret the UV-Vis results and to quantitatively estimate the light absorption coefficient of each optical coupling material during each temperature trial, we average the results of each temperature trial from 350 nm to 650 nm. The results of this are shown in Figure 6. The smallest average value of light absorption coefficient was obtained at 10 or 20 °C for OG0010, BC-630, and OC431A-LVP while at −10 °C for BLUESIL V-788. The maximum average value of light absorption coefficient was achieved at −20 °C for OG0010, 40 °C for OC431A-LVP, 0 °C for BLUESIL V-788, and 30 °C for BC-630. Average light absorption coefficient was smallest around middle temperatures and larger at temperature extremes.

**Figure 7:**
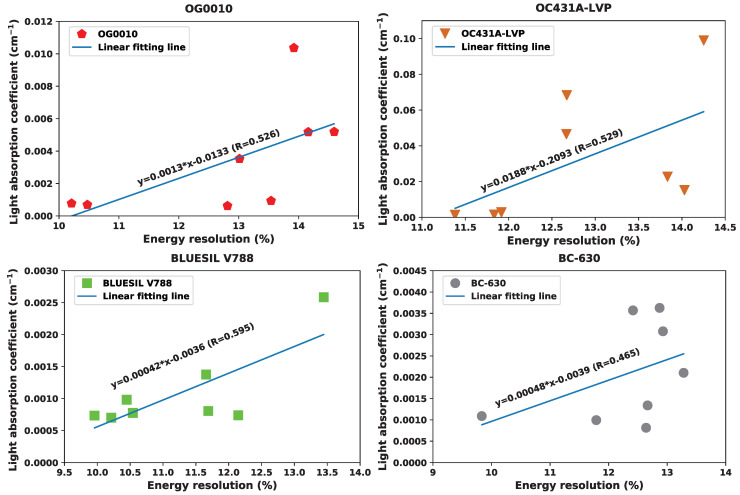
The relationships between the light absorption coefficient and the energy resolution for all four optical coupling materials.

Additionally, the results of the temperature-averaged light absorption coefficient were compared with the energy resolution at each temperature for each optical coupling grease, shown in Figure 7. A simple linear regression was performed on this data, and the R-squared was computed. All four of the optical coupling greases have a positive correlation between the light absorption coefficient and the value of the energy resolution. Further, the R-squared value is close or over 0.5 in all cases, which indicates a moderate effect size.

## 4. Discussion

Any material perturbations impacting the refractive index, light absorption coefficient, quality of surface coverage, thickness of the coupling layer, or transparency to optical photons will translate into performance shifts—whether large or small—for radiation detectors. Temperature exposure is one such mechanism which may incite either material changes in the optical coupling material itself or coverage changes in the surface interfaces. Throughout testing, no partial or total loss of coupling was observed, i.e., surface-to-surface coverage remained intact by means of the coupling gel.

First, in Figure 2, note that the performance of all coupling materials does indeed change with temperature after the 2-h exposure and subsequent return to room temperature. The shifts in energy resolution are nonnegligible as the variance encompassed by the full set of measurements for a given coupler cannot be explained by the variance seen at a single temperature point for that coupler. After the material is applied to the detector and exposed to temperatures, a clear performance shift is evident. Importantly, a change in energy resolution is apparent for all optical coupling materials (except BLUESIL V-788) around room temperature—between 10 and 30 °C. This temperature range may be commonly seen by all types of detectors and highlights the importance of this study. Perturbations in energy resolution could lead to the inability to resolve different photopeaks, leading to the inability to accurately identify present radioisotopes. Changes in photopeak width can also adversely affect spectrum-based analysis models expecting consistent energy channels to be occupied with photopeak or background counts.

We can additionally observe in Figure 2 that all the 20 °C (near room temperature, 23 °C) trials had similar performance with small error bars. Considering the identical storage conditions, similar ages, and similar indices of refraction of the tested materials, this is reassuring from a methodological standpoint and important to note from an experimental standpoint. The scintillation crystal and SiPM were exposed to the −20 to 50 °C temperature range (hottest to coldest) four times—once for each series of coupler tests. The 20 °C trial is then an indicator of SiPM and scintillation crystal health. With the data at hand, there is no evidence of scintillation crystal or SiPM performance change due to thermal shock.

The observable trends present in Figure 2 also show a different temperature response between BLUESIL V-788 and the remaining couplers. OG0010, OC431A-LVP, and BC-630 generally have worse energy resolution at the extreme temperatures and better performance at median temperatures. BLUESIL V-788, on the other hand, has better energy resolution at the extreme temperatures than at median temperatures. This phenomenon may be due to a difference in additives to the greases or other material difference. The UV-Vis experiment helps shed some light on this phenomenon.

Figure 5 and Figure 6 show the results of light absorption coefficient from the UV-Vis experiments. The larger the light absorption coefficient is, the more the induced optical photons from the scintillation crystal would be absorbed (or attenuated) by the optical coupling material itself. Large light absorption leads to less light reaching the SiPM and yields poorer energy resolution. We observed that the optical coupling greases have relatively large absorption coefficients at −20, 30, 40, and 50 °C for BC-630, OC431A-LVP, and OG0010, which generally corresponded to the poor energy resolution performance seen in Figure 2 at these temperatures. This correlation, however, can fully explain neither the variance observed in energy resolution over this temperature range nor the variance at single temperature points. For example, these three optical coupling greases—particularly OG0010 and BC-630—experienced a substantial performance change between 10 and 20 °C despite the light absorption coefficient remaining approximately constant. The linear modelling, shown in Figure 7, displays this relationship more quantitatively. With an R-squared of approximately 0.5 and positive correlation between light absorption coefficient and energy resolution, a relationship between the two is evident but cannot be used to explain all the variance observed in the data. Therefore, while the temperature-induced variance in the light absorption coefficient can explain some performance shifts, it is only one in potentially many reasons causing the variance in energy resolution performance of the optical coupling greases.

For each coupler, recommended temperature ranges are offered (as seen in Table 1) for preserving intended performance. Manufacturers, however, have different criteria when presenting these ranges, so they are difficult to directly compare. In addition to this, it can be unclear what performance metric is used to establish the recommended range. For example, Elkem notes in its technical data sheet (TDS) “Maximum Clarity with BLUESIL V-788 is between 65 and 80 °F (18 to 27 °C).” [13]. In comparison, the Smartgel product brochure [18] states that “SmartGels [OC431A-LVP] are designed to withstand wide temperature excursions (−65 °C to ≥ 200 °C)." The TDS does not provide more detailed information. CAEN support provided information on the OG0010 optical grease: “It [OG0010] should be stored at temperatures below 26 °C, preferably below 5 °C. Retains clarity and fluid property down to −60 °C." Finally, SAINT-GOBAIN support states that BC-630 has “maximum light transmission between 18 and 27 °C.” BLUESIL V-788 and BC-630 ranges appear to be based exclusively on light transmissivity, while OC431A-LVP and OG0010 ranges are concerned with general fluid properties. Both are legitimate ways of defining the recommended usage range, but they no doubt result in different performance expectations. Having both for the same coupler may be helpful in selecting the right optical coupler for the task at hand.

We would recommend OC431A-LVP for tasks where large temperature changes are expected because it had the smallest variance over the −20 to 50 °C testing range. For near room-temperature tasks, we recommend BC-630 because it had the most stable performance above 20 °C. This recommendation is supported by the manufacturer recommended temperature ranges.

## 5. Conclusions

This work serves to systematically evaluate the temperature performance of a detector system with several optical coupling greases. When evaluating the whole-detector performance, the silicone greases OG0010, OC431A-LVP, and BC-630 had similar performance over the selected range whereas BLUESIL V-788 differed. All tested coupling materials, however, did display changes in performance after temperature exposure. OC431A-LVP had the smallest variance over the −20 to 50 °C testing range, while BC-630 had the most stable performance above 20 °C. In the second applied testing method, the light absorption coefficient of the optical coupling materials was isolated for temperature exposure via a UV-Vis spectra measurement. The test results of this method show that temperature exposures do change the light absorption coefficient of optical coupling greases, which can ultimately change the energy resolution performance of each optical coupling grease. While optical greases are a popular coupling material, they are not the only type available. Silicone rubber optical interfaces and curing optical cement may be more appropriate for long-term use cases. However, the same concerns exist for these variants and similar studies should be conducted. On a similar note, this is a performance-based study of the full-detector performance, and we do not offer physiochemical analysis of the coupling greases. Future material-centric studies can be conducted to include this type of analysis. We conclude that temperature does impact the performance of the optical coupling grease in radiation detectors. 

## Figures and Tables

**Table 1:** Reported properties of the optical coupling agents: values marked with * were acquired from correspondence with technical support staff at their respective suppliers. Densities and indices of refraction without available temperature or wavelength information, respectively, do not specify these values.

Agent	Supplier	Index of Refraction	Density (g/cc)	Temperature Range (°C)
SMARTGEL OC431A-LVP	Nye Lubricants	1.4988 @ 402 nm [17]	4.4 @ 25 °C [17]	−65 to 200 [18]
OG0010 Optical Grease	CAEN SpA	1.465 *	1.03 *	−60 to 26 *
BLUESIL V-788 [13]	Elkem	1.463	1.06	18 to 27
BC-630	Saint-Gobain Crystals	1.465 [19]	1.04 @ 25 °C [20]	18 to 27 *

**Table 2:** Summary of the properties of Lutetium-yttrium oxyorthosilicate (LYSO) scintillation crystal.

Crystal	Dimensions	Density	Energy Resolution	Index of Refraction	Effective Z Number
LYSO	3 × 3 × 15 mm${}^{3}$	7.1 g/cc [16]	8.2% @ 662 keV [21]	1.81 @ 420 nm [16]	63.5 [21]

**Table 3:** Sample variance of room-temperature energy resolution tests compared to the full set sample variance.

Agents	Room Temp Variance	Full Set Variance
OG0010	0.3	3.0
OC431A-LVP	0.3	1.5
BLUESIL V-788	0.1	1.7
BC-630	0.6	1.7

**Table 4:** Count statistics for the total counts over all trials for each coupling agent.

Coupling Agent	Average Total Counts	Standard Deviation (Counts)	Standard Deviation (% of Average)
OC431A-LVP	5.3 × 10${}^{6}$	0.1 × 10${}^{6}$	1.9
OG0010	5.63 × 10${}^{6}$	0.09 × 10${}^{6}$	1.60
BLUESIL V-788	5.52 × 10${}^{6}$	0.03 × 10${}^{6}$	0.543
BC-630	5.77 × 10${}^{6}$	0.04 × 10${}^{6}$	0.693

## Abbreviations

The following abbreviations are used in this manuscript:

SiPM	silicon photomultiplier
LYSO	Lutetium-yttrium oxyorthosilicate
ANSI	American National Standards Institute
TDS	Technical Data Sheet
UV-Vis	ultraviolet-visible
